# Integrative utilization of genomic resources for improved phylogenetic resolution in Sonerileae (Melastomataceae)

**DOI:** 10.1002/ajb2.70216

**Published:** 2026-06-10

**Authors:** Luo Chen, Marie Claire Veranso‐Libalah, J. Peter Quakenbush, Ying Liu, Fabián A. Michelangeli, Diego F. Morales‐Briones, Gudrun Kadereit

**Affiliations:** ^1^ Prinzessin Therese von Bayern‐Lehrstuhl für Systematik, Biodiversität & Evolution der Pflanzen, Ludwig‐Maximilians‐Universität München Munich 80638 Germany; ^2^ Botanische Staatssammlung und Botanischer Garten München, Staatliche Naturwissenschaftliche Sammlungen Bayerns (SNSB) Munich 80638 Germany; ^3^ Senckenberg Research Institute and Natural History Museum Frankfurt am Main 60325 Germany; ^4^ Department of Biology Grand Valley State University Allendale 49401 MI USA; ^5^ School of Ecology, Sun Yat‐sen University Shenzhen 518107 China; ^6^ Center for Biodiversity & Evolution, New York Botanical Garden Bronx 10458 NY USA

**Keywords:** data integration, deep genome skimming, genome skimming, Melastomataceae, phylogenomics, RNA‐Seq, Sonerileae, target enrichment

## Abstract

**Premise:**

Advances in new next‐generation sequencing (NGS) methods have revolutionized phylogenetics, yet challenges remain in effectively utilizing data from a wide range of sources. A well‐resolved and broadly sampled phylogeny for Sonerileae, the second‐largest tribe in Melastomataceae, is still lacking, hindering our understanding of its phylogenetic relationships and evolutionary history.

**Methods:**

We combined target enrichment, genome skimming, RNA‐Seq, and deep genome skimming (DGS) data to reconstruct the phylogenetic relationships within Sonerileae. We sampled 184 accessions representing 41 of the 47 genera in the tribe, including new sequences obtained with Hyb‐Seq and DGS, and publicly available sequences. A newly developed reference comprising 5626 loci was employed to integrate data from different sources.

**Results:**

Our analyses produced a well‐supported, near‐comprehensive phylogeny for the tribe, outperforming previous and current studies that used only target enrichment probe sets as the reference for loci extraction. For the long‐unresolved Asian Sonerileae, our phylogeny is comparable to that of a study focused specifically on that group, where a draft genome was required to identify orthologs.

**Conclusions:**

Our analyses demonstrate that using an inclusive reference file enables the integration of genomic data generated with different sequencing strategies. We provide an effective approach for combining heterogeneous data for phylogenomic analysis.

The increasing availability of next‐generation sequencing (NGS) data has significantly changed the field of phylogenetics in the past 10 years (Guo et al., 2023). In response, a variety of sequencing strategies have been established for phylogenetic studies, including target enrichment, genome skimming, deep genome skimming (DGS), Hyb‐Seq, and RNA‐Seq. Among these, target enrichment, or target capture, is currently one of the most popular approaches for plant phylogenomics. It selects targeted genomic regions before sequencing, providing a cost‐effective approach to obtain hundreds to thousands of loci (Mandel et al., 2014). This method has proven effective across various taxonomic scales, from population‐level studies (e.g., Villaverde et al., 2018; Jiménez‐Mena et al., 2022) to higher level phylogenetics (e.g., Benítez‐Villaseñor et al., 2023). A universal probe set targeting 353 nuclear loci across angiosperms, the Angiosperms353 kit (Johnson et al., 2018), exemplifies this approach and has been successfully used to resolve numerous flowering plant lineages, including species sampled from herbarium specimens over 200 years old (Zuntini et al., 2024).

Another commonly used sequencing strategy is genome skimming, which was originally developed as a low‐coverage method to capture high‐copy genomic regions (Straub et al., 2012). It has been widely applied in organellar genome sequencing due to the high copy numbers of plastid and mitochondrial genomes (Twyford and Ness, 2017; Trevisan et al., 2019). In addition to these high‐copy regions, the potential to recover low‐copy nuclear loci from genome skimming data was noted in the original study and has since been explored in subsequent studies (Straub et al., 2012; Blischak et al., 2014; Pezzini et al., 2023; Pouchon and Boluda, 2023).

More recently, Liu et al. (2021) demonstrated that with sufficient sequencing depth (at least 10× in their data set), numerous high‐quality single‐copy nuclear genes could also be assembled from genome skimming data. They termed this approach deep genome skimming, which enables the assembly of numerous single‐copy nuclear genes and complete plastomes, mitochondrial genomes, and often the entire ribosomal DNA sequences without the need for target enrichment. This approach has since been applied to various lineages of organisms (e.g., Liu et al., 2021; Yang et al., 2023; Hu et al., 2024; Quattrini et al., 2024; Niu et al., 2025) and is particularly effective for groups with relatively small genomes (e.g., ~500 Mb, Liu et al., 2021; ~900 Mb, Yang et al., 2023; ~500–750 Mb, Quattrini et al., 2024), but can also be applied to relatively large genomes as long as a sufficient number of reads are generated (e.g., ~3 Gb in Hu et al., 2024; ~1.66–2.32 Gb in Niu et al., 2025).

Hyb‐Seq is a sequencing strategy that combines target enrichment and genome skimming. It was initially proposed for sequencing targeted genomic regions using on‐target reads and for recovering high‐copy genomic regions through off‐target reads from the enriched library (Weitemier et al., 2014), making it essentially interchangeable with target enrichment. The term can also refer to an approach that involves sequencing both enriched (target enrichment) and unenriched (genome skimming) libraries (Villaverde et al., 2018; Dodsworth et al., 2019) to improve recovery of high‐copy regions of the genome; here, we use this definition.

RNA‐Seq, or transcriptome sequencing, was originally developed for transcriptome profiling (Wang et al., 2009), but it also has great utility in phylogenetic studies (Oakley et al., 2012; Wickett et al., 2014; Leebens‐Mack et al., 2019). When orthologs are accurately identified, the phylogenetic results are comparable to those obtained from whole‐genome sequencing (Cheon et al., 2020). The challenge with RNA‐Seq is that total RNA extraction requires fresh tissue, flash‐frozen tissue stored at –80°C (Chomczynski and Sacchi, 2006), or tissue preserved in reagents such as RNALater (Yockteng et al., 2013). Although a few studies have successfully extracted RNA from silica‐dried plant tissues (Chen et al., 2022; He et al., 2022; Ruiz‐Vargas et al., 2024), this approach has not been widely tested across different groups and remains impractical for herbarium specimens.

Despite the potential to significantly improve data (re‐)utilization and the general importance of integrative data use, no one has yet explored the compatibility of these different types of genomic data when combined into a single phylogenomic inference or optimizing the strategy. A common strategy is to recover loci targeted for enrichment from RNA‐Seq and DGS data (Bossert et al., 2019; Wang et al., 2024). However, this strategy uses only the subset of reads corresponding to targeted loci, leaving much of the remaining information in the RNA‐Seq and DGS data underutilized. As more data are generated by different research groups using various sequencing strategies, it becomes increasingly necessary to identify an effective approach to integrating all NGS data sets available for a given group of organisms while maximizing information in the sequencing data.

Large, species‐rich plant groups that have been studied for a long time by multiple research groups provide good examples of the challenges of data integration because the available sequence data sets are often heterogeneous. The Melastomataceae are one of the 10 largest families of flowering plants, comprising 5858 species across 173 genera in three subfamilies, one of which is further divided into 21 tribes (Ulloa Ulloa et al., 2022). Sonerileae (Figure 1) is the second‐largest tribe within Melastomataceae, containing 47 genera and around 1080 species distributed throughout the tropics (Lin et al., 2022; Liu et al., 2022; Veranso‐Libalah et al., 2023; Liu et al., 2024; Quakenbush et al., 2025; Zhuang et al., 2025). The generic delimitations within Sonerileae have long been unresolved because many of the morphological traits used are homoplasious and sampling for molecular phylogenetic studies has been limited (Zhou et al., 2019a, 2019b). A recent phylogenomic study (Zhou et al., 2022) on the Asian Sonerileae, which included samples of most recognized Asian genera and reconstructed their phylogeny using DGS data, identified 34 lineages within the Asian Sonerileae. Based on the results of this study, the genus *Perilimnastes* was reinstated (Liu et al., 2024), and *Nephoanthus* was newly described (Lin et al., 2022). The authors further expanded sampling for the *Cyphotheca*‐*Plagiopetalum*‐*Sporoxeia* clade and described three new genera, i.e., *Chiehchenii*, *Neophyllagathis*, and *Sporocyphoxeia*, based on phylogenetic and morphological evidence (Zhuang et al., 2025). In another phylogenomic study (Quakenbush et al., 2025) that focused on the systematics of fleshy‐fruited Sonerileae using mostly target enrichment data, the fleshy‐fruited Sonerileae were found to be nonmonophyletic. Within this group, *Pachycentria* and *Plethiandra* were recovered as nested within *Medinilla* and were consequently synonymized to maintain the monophyly of *Medinilla*. However, African and neotropical lineages remain underrepresented in both studies. A separate phylogenomic study (Maurin et al., 2021) using Angiosperms353 on the Myrtales included some African and neotropical Sonerilean genera, but the sampling was limited because the study focused on familial relationships. While the Kew Tree of Life Explorer (Baker et al., 2021) is gradually incorporating more species, the sampling for Sonerileae remains limited. Consequently, our understanding of the relationships among African and neotropical Sonerileae is still primarily based on a Sanger sequence‐based phylogeny (Veranso‐Libalah et al., 2023), in which neotropical genera and the African endemic *Benna* were identified as successive sister lineages to the remaining Sonerileae, which is divided into an African‐Malagasy endemic clade and a predominantly Asian clade. Based on this study, the genus *Mendelia* was described, and *Bourdaria* was reinstated.

**Figure 1:**
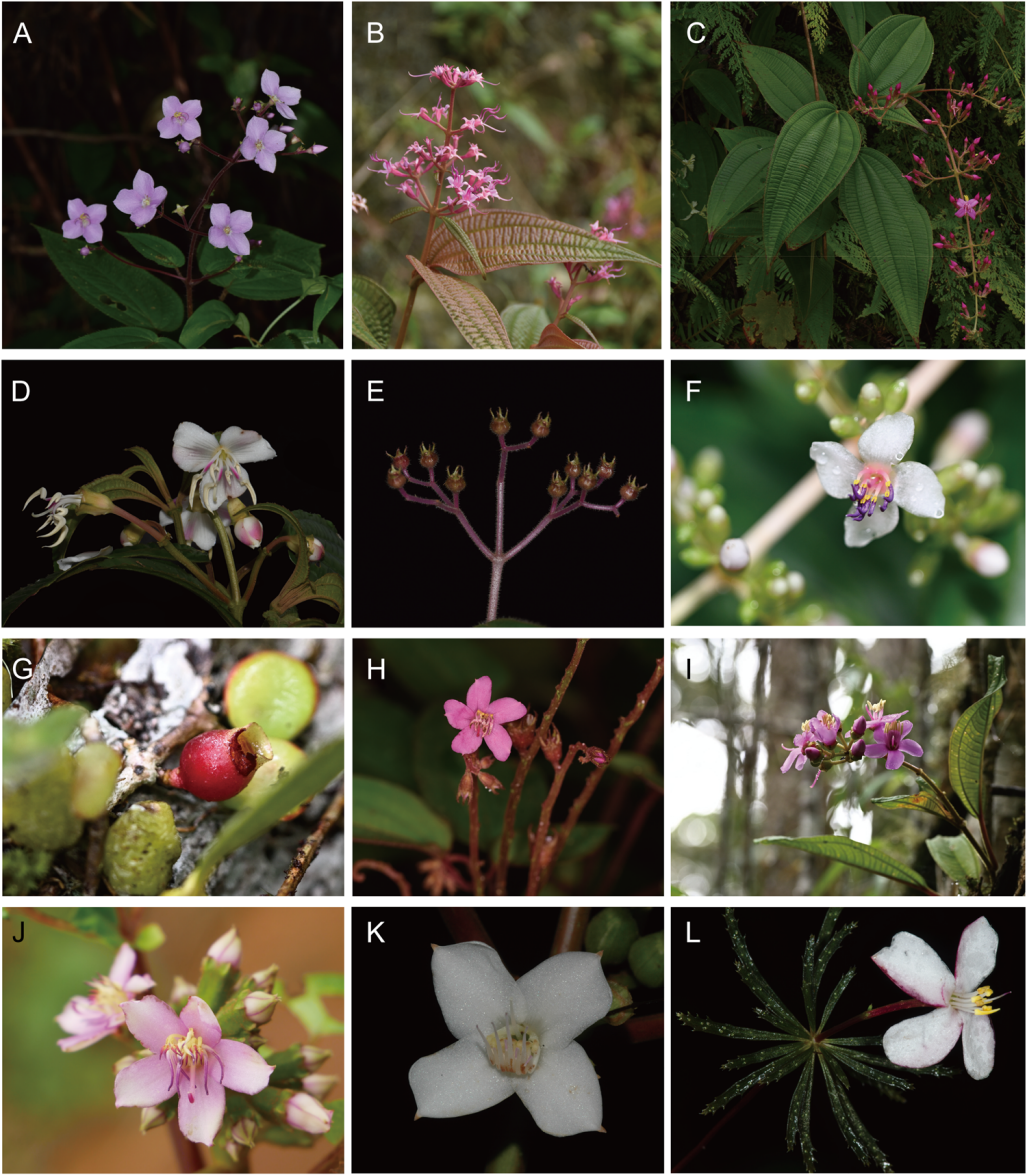
Representatives of the major clades of Sonerileae. (A) *Bredia hirsuta*, (B) *Blastus cavaleriei*, (C) *Oxyspora paniculata*, (D) *Cyphotheca montana*, (E) *Scorpiothyrsus shangszeensis*, (F) *Medinilla pendula*, (G) *Medinilla sedifolia*, (H) *Mendelia mollis*, (I) *Gravesia laxiflora*, (J) *Dicellandra barteri*, (K) *Opisthocentra clidemioides*, (L) *Tryssophyton merumense*. Photo credits: A–E, Ying Liu; F–I, Luo Chen; J, Andreas Gröger; K, Mario Terra; L, John L. Clark.

A well‐represented and well‐supported phylogeny is crucial for understanding the relationships among the African, Asian, and neotropical Sonerileae and the evolutionary history of the tribe, including its biogeography and trait evolution. A large amount of sequence data for Sonerileae has already been published. Therefore, the aim of our current study is to mobilize and reuse these published data, extract loci, and integrate them into a new analysis, adding previously missing or underrepresented taxa. To reach this aim, we developed a new reference comprising 5626 loci for Sonerileae based on genomic and transcriptomic data. Using this integrative reference, we reconstructed a near‐comprehensive phylogeny for the tribe by combining previously and newly generated Hyb‐Seq and DGS data with all available NGS data, including RNA‐Seq, DGS, target enrichment from different probe sets, Hyb‐Seq, and annotated genomes. A primary goal of our study was to assess how far this approach advances our understanding of the systematics and evolution of Sonerileae.

## MATERIALS AND METHODS

### Sampling and sequencing

We included 184 accessions, 179 of which belong to Sonerileae, representing 41 of the 47 genera, which include four of the six neotropical genera, all nine afrotropical endemic genera, 27 of the 31 Asian/Oceanian endemic genera, and the only afrotropical‐Asian disjunct genus within the tribe, *Medinilla*, for which we sampled all alliances recognized by Quakenbush et al. (2025). All 34 Asian lineages identified by Zhou et al. (2022), supported by both the single‐copy ortholog (SCO) and genomic SNP data sets in that study, were included. Additionally, we selected four *Melastoma* and one *Osbeckia* species from the tribe Melastomateae as outgroups. To obtain a near‐complete genus‐wide phylogeny, we incorporated a variety of data types: Among the 184 accessions, 73 were newly sequenced, while sequence data for the remaining 111 accessions were obtained from public resources: RNA‐Seq data for 15 accessions from the NCBI Sequence Read Archive (SRA) and China National GeneBank (CNGB), annotated genomes for two accessions from the National Genomics Data Center (NGDC), Angiosperms353 target enrichment data for 16 accessions from the European Nucleotide Archive (ENA), and DGS data for 78 accessions from the SRA. Details on the vouchers and accession numbers are provided in Appendix S1.

Of the 73 newly sequenced accessions, 70 were sequenced using Hyb‐Seq and the remaining three using DGS. For the 70 accessions sequenced with Hyb‐Seq, target enrichment data for 55 had previously been published (Quakenbush et al., 2025), to which genome skimming data were now added. In addition, complete Hyb‐Seq data were newly generated for 15 accessions. DNA extraction, library preparation, target enrichment, and next‐generation sequencing were performed as described by Quakenbush et al. (2025). The Melastomataceae probes (Jantzen et al., 2020) were used to capture 384 loci during target enrichment, while ~1 Gb of data were generated during genome skimming. For the DGS samples, DNA was extracted following Quakenbush et al. (2025). Library preparation and sequencing for these samples were performed at Novogene Europe (Cambridge, UK). Paired‐end reads were generated on the Illumina NovaSeq X Plus system (San Diego, CA, USA), with a requested data yield of 6 GB per sample.

### Nuclear reference construction

**Figure 2:**
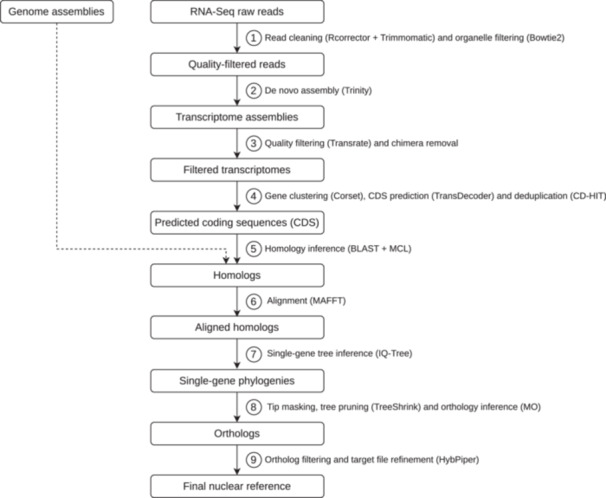
Workflow for constructing the nuclear reference used in this study.

To effectively integrate the various data in this study, we developed a new nuclear reference sequence file using 16 RNA‐Seq samples and the annotated genome for *Melastoma candidum* (Zhong et al., 2023) and *Barthea barthei* (Huang et al., 2024). These 18 samples included five species from Melastomateae, 12 species from Sonerileae, and one species from Miconieae. The pipeline described here (Figure 2) largely follows that of Yang and Smith (2014) and Morales‐Briones et al. (2021) (https://bitbucket.org/yanglab/phylogenomic_dataset_construction). Raw reads of the RNA‐Seq data were processed to correct random sequencing errors using Rcorrector v1.0.6 (Song and Florea, 2015), remove adapters and low‐quality sequences with Trimmomatic v0.39 (Bolger et al., 2014), and filter organelle reads using Bowtie2 v2.4.5 (Langmead and Salzberg, 2012). De novo transcriptome assembly was performed using Trinity v2.13.2 (Grabherr et al., 2011), then quality was assessed and low‐quality transcripts were filtered out using Transrate v1.0.3 (Smith‐Unna et al., 2016), and chimeric transcripts were removed using a blast‐based method (Yang and Smith, 2013). Filtered transcripts were clustered into putative genes using Corset v1.09 (Davidson and Oshlack, 2014) with the longest transcript kept. Coding sequences (CDS) were identified using TransDecoder v5.3.0 (Haas et al., 2013). Before homology inference, transcript redundancy was further reduced using CD‐HIT v4.8.1 (Fu et al., 2012). Homology was inferred by first performing all‐by‐all BLASTN v2.10.0+ (Camacho et al., 2009) on the 16 transcriptomes and two annotated genomes and then using MCL (van Dongen and Abreu‐Goodger, 2012) to group transcripts into clusters, with the hit fraction cutoff set to 0.4 and inflation values to 1.4. The resulting 12,497 clusters were aligned using MAFFT v7.525 with the algorithm “localpair” (Katoh and Standley, 2013). The aligned clusters were trimmed using Phyutility v2.7.1 (Smith and Dunn, 2008) to discard columns with more than 90% missing data. Single homologous gene trees were inferred using IQ‐Tree v2.3.5 (Minh et al., 2020), with ModelFinder (Kalyaanamoorthy et al., 2017) employed to select the best substitution model. The raw single trees were trimmed by masking monophyletic and paraphyletic tips that belong to the same species, retaining the tip with the most unambiguous characters in the trimmed alignment. Spurious long branches were then pruned with TreeShrink v1.3.9 (Mai and Mirarab, 2018). The MO (monophyletic outgroup) method (Yang and Smith, 2014) was employed to infer orthology. Gene trees without taxon duplication were retained as one‐to‐one orthologs if they had monophyletic outgroups. For trees with taxon duplication, only those with monophyletic, nonrepetitive outgroups were rerooted, and the orthologous subtree with the highest taxon number was retained. The resulting orthologs with fewer than 12 taxa were discarded. We also filtered gene trees if the Sonerileae or Melastomateae taxa did not form a clade. The corresponding fasta files of the final 5639 gene trees were written, combined, and checked with Hybpiper (Johnson et al., 2016) command check_targetfile and processed with command fix_targetfile to correct reading frames and filter sequences with low‐complexity regions, resulting in 5626 retained loci. To assess the overlap among the newly developed 5626‐locus reference, the Melastomataceae probe set, and the Angiosperms353 probe set (specifically a tailored mega353 file for Myrtales, see McLay et al. (2021) and Chen et al. (2023)), we built a local BLAST database from the new reference using the application makeblastdb (Camacho et al., 2009) and searched the sequences against the other two references using the application blastn with default settings.

### Reads processing, assembly, and reference loci recovery

Reads processing, assembly, and reference loci recovery were conducted using Captus v1.2.0 (Ortiz et al., 2023 [preprint]). Since the sequences from the RNA‐Seq samples and annotated genomes used to construct the new reference were already available, these data were excluded from this step. Target enrichment and skimming data generated by Hyb‐Seq for this study were first merged. Raw reads from the DGS, Angiosperms353 target enrichment, and Hyb‐Seq data were trimmed using BBDuk from BBTools (Bushnell, 2022). For the Angiosperms353 data, trimming followed the default settings in Captus, whereas the DGS and Hyb‐Seq data were trimmed with more stringent parameters (“‐‐trimq 20 ‐‐maq 20”). The cleaned reads were then de novo assembled into contigs using MEGAHIT (Li et al., 2015) with default parameters. Reference loci were recovered from the assemblies using Scipio (Hatje et al., 2011) with minimum percentage identity (option ‐‐nuc_min_identity) set to 80 and coverage (option ‐‐nuc_min_coverage) to 50. The newly constructed reference was used to recover loci. The percentage of recovered sequence length relative to the reference sequence was calculated in Captus and visualized using a custom Python script (ChatGPT [OpenAI, 2026] was used to assist with coding for this script. The generated code was critically reviewed, tested, and revised by the authors before use.). To maximize data use, loci in the Melastomataceae probe set but absent from the new reference were also used to recover loci.

To compare the performance of the new reference and the Melastomataceae probe set, we recovered the 396 loci of the Melastomataceae probe set (Jantzen et al., 2020; Dagallier and Michelangeli, 2024) across all 184 assemblies, including the RNA‐Seq sequences.

### Orthology inference

The recovered reference loci were retrieved using Captus for further analysis. For the new reference data set, reference sequences from Sonerileae and Melastomateae were added to each cluster. This step was not necessary for the 396‐locus data set. Each cluster was aligned and trimmed using MAFFT and Phyutility, following the same procedures as in the construction of the nuclear reference. Sequences shorter than 100 bp or with more than 90% missing sites were discarded. Single gene trees were inferred using IQ‐Tree with the GTRGAMMA substitution model, and branch support was assessed using 1000 ultrafast bootstrap replicates. Monophyletic and paraphyletic tips were pruned, spurious long branches were trimmed, and orthology inference using the MO method followed the same approach as in nuclear reference construction. The five sampled Melastomateae species were set as outgroups, and only trees with at least 19 tips were retained. We calculated the occupancy of each taxon in the final orthologs for both the new reference and the 396‐locus data set.

### Phylogenetic analyses

The orthologs were realigned, trimmed, and gene trees were inferred using the same methodology as in the orthology inference. Species trees were then reconstructed using ASTRAL‐IV v1.19.4.5 (Zhang et al., 2018; Zhang and Mirarab, 2022; Tabatabaee et al., 2023). The tree based on the new reference is referred to as ST1, and the tree based on the 396‐locus data set is referred to as ST2. We also inferred a species tree (ST3) for the 16 RNA‐Seq samples and two annotated genomes used for reference construction, based on the 5639 orthologs. The phylogenetic results were visualized using ggtree 3.10.0 (Yu et al., 2017) in R version 4.3.2 (R Core Team, 2023).

### Assessing gene tree conflicts

The quartet‐based method, quartet sampling (QS) (Pease et al., 2018), was used to assess the level of discordance among gene trees. For a given phylogeny, any internal branch can divide the tree into four sets of taxa. Quartet sampling repeatedly and randomly selects one taxon from each set and evaluates the likelihoods of all three possible topologies: one that is concordant and two that are discordant with the given phylogeny, based on the given sequence data. The species tree ST1 and an alignment generated by concatenating gene alignments used for gene tree inference with AMAS (Borowiec, 2016) were used as input. For each internal branch, 6000 replicates were performed, and the option ‐‐genetrees was used to enable partitioned analysis. Likelihood evaluations were carried out using RaxML (Stamatakis, 2014). Quartet concordance (QC), quartet differential (QD), and quartet informativeness (QI) scores were generated. The QS results were visualized using an R script developed by Liu et al. (2025).

## RESULTS

### New reference and sequence recovery

The newly developed reference comprises 5626 loci represented by a total of 76,834 sequences. The length of each locus ranges from 300 to 11,127 bp, with an average of 1419 bp and a median of 1275 bp. According to the BLAST results, 176 of these loci are putatively shared with the Angiosperms353 loci, and 221 are putatively the same as those in the Melastomataceae probe set. For details of the matching loci, see Appendix S2.

**Figure 3:**
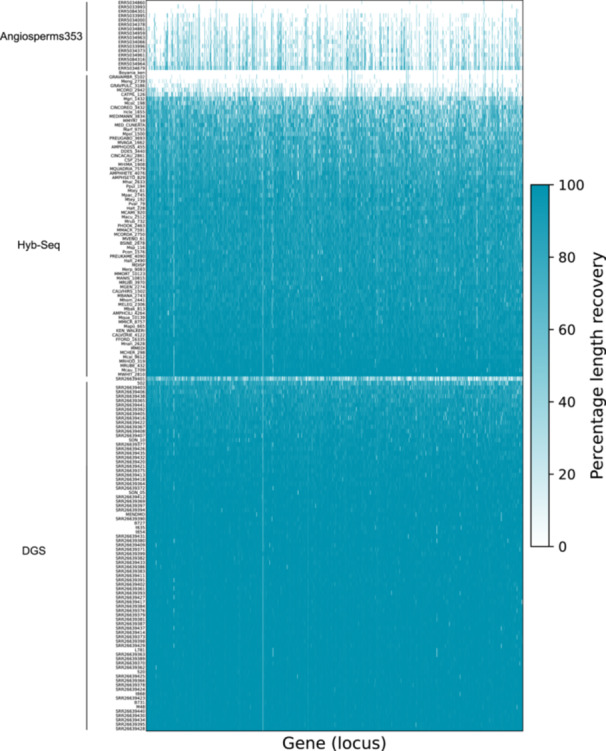
Heat map of percentage length recovery of new reference loci across samples sequenced with Angiosperms353 probe set, Hyb‐Seq, and deep genome skimming (DGS).

Of the 5626 target loci, 52–5625 loci were recovered per sample with Captus (Figure 3; Appendix S3). Sequence recovery differed among data sets: Angiosperms353 samples recovered 52 to 2353 loci (average 1082, median 1180), Hyb‐seq samples recovered 74 to 5622 loci (average 5024, median 5578), and DGS samples recovered 3883 to 5625 loci (average 5586, median 5619).

### Orthology inference

**Figure 4:**
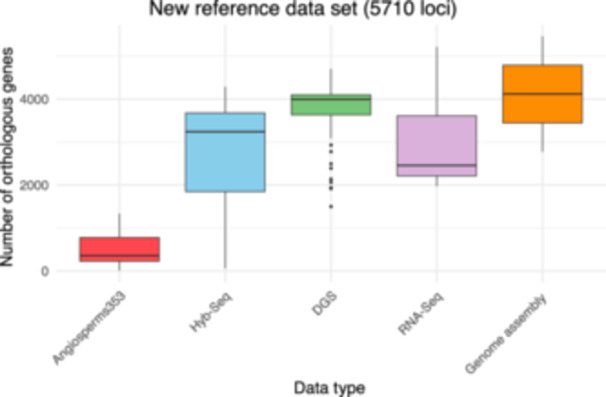
Distribution of orthologs per taxon by different sequencing strategies in the final 5710 orthologs from the new reference data set.

Both the newly developed reference and the Melastomataceae probe set were used for phylogenetic analysis. We obtained 5710 orthologs from the new reference data set, including 106 orthologs derived from the 175 non‐overlapping loci from the Melastomataceae probe set, while 225 orthologs were retrieved from the 396‐locus data set. We calculated the frequency of taxa in the final sets of orthologs. The representation across different sequencing strategies for the new reference data set was as follows (Appendix S4; Figure 4): Annotated genome samples had 2774 to 5459 loci represented (average 4117); RNA‐Seq samples had 1972 to 5214 loci (average 3024, median 2456); Hyb‐Seq samples had 62 to 4287 loci (average 2775, median 3241); Angiosperms353 samples had 10 to 1336 loci (average 483, median 359); and DGS samples had 1499 to 4703 loci (average 3738, median 3994). The representation across different sequencing strategies for the 396‐locus data set is listed in Appendix S5 (see also Appendix S6).

### Phylogenetic analyses

**Figure 5:**
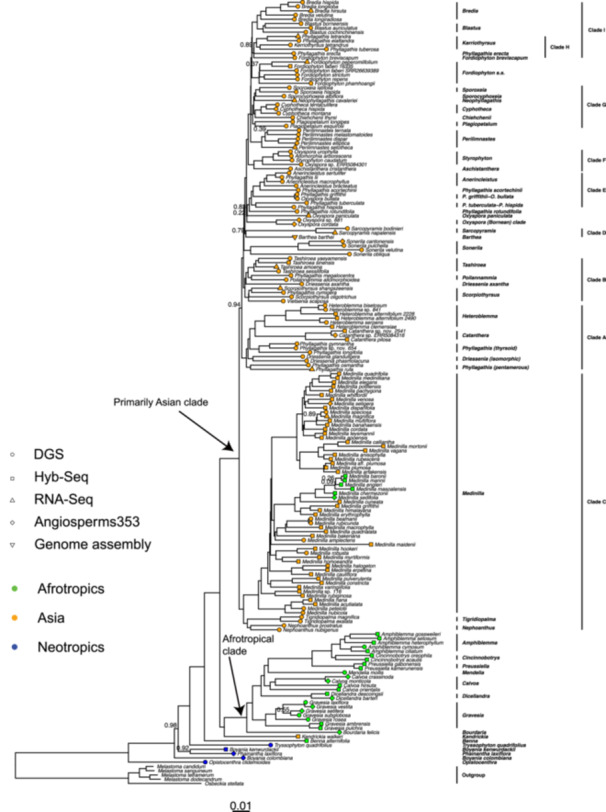
ASTRAL species tree ST1 inferred from 184 taxa and 5710 orthologs obtained from the new reference data set, showing the relationships within Sonerileae. Local posterior probabilities (LPP) are shown above the branches where LPP < 1.

The species tree ST1 inferred from the new reference data set is well resolved with most nodes fully supported (LPP = 1) (Figure 5). The neotropical taxa (*Opisthocentra*, *Boyania colombiana*, *Boyania kenwurdackii* + *Phainantha*, *Tryssophyton*) are successive sisters to the rest of the tribe, followed by the African endemic *Benna*, and then *Kendrickia* (an Asian genus) plus the afrotropical endemic clade. After these, the tree revealed a primarily Asian clade, with only one dispersal back to the Afrotropics within the diverse genus *Medinilla*. Within the afrotropical clade, *Bourdaria* is sister to all other genera, followed by a grade composed of *Gravesia*, *Dicellandra*, *Calvoa*, *Mendelia*, and *Preussiella*. *Amphiblemma* is sister to *Cincinnobotrys* (Figure 5).

Within the primarily Asian clade, all 34 sampled lineages and nine clades (clades A–I) identified by Zhou et al. (2022) were recovered. The sampled taxa formed two major clades. The first corresponds to clade C, which includes *Medinilla*, *Tigridiopalma*, and *Nephoanthus* (formerly the *Phyllagathis prostrata* clade). The second major clade contains the remaining genera, starting with a lineage largely corresponding to clade A, now incorporating *Catanthera*. Clade A includes *Heteroblemma*, *Catanthera*, the *Phyllagathis* (thyrsoid) clade, the *Phyllagathis* (pentamerous) clade, and the *Driessenia* (isomorphic) clade. The next branching clade is clade B, comprising *Tashiroea*, *Poilannammia*, *Scorpiothyrsus*, and *Driessenia axantha*. Next is *Sonerila*, then clade D, which contains *Sarcopyramis* and *Barthea*. The next clade includes clade E (comprising *Anerincleistus*, *Phyllagathis scortechinii*, *Phyllagathis griffithii*‐*Oxyspora bullata*, and *Phyllagathis tuberculata*‐*P. hispida*), *Phyllagathis rotundifolia*, *Oxyspora paniculata*, and the *Oxyspora* (Bornean) clade. This clade is followed by clade F (which includes *Aschistanthera* and *Styrophyton*), *Perilimnastes* (formerly the *Phyllagathis* [raphides] clade), then clade G (consisting of *Sporoxeia*, *Sporocyphoxeia*, *Neophyllagathis*, *Cyphotheca*, *Chiehchenii*, and *Plagiopetalum*), then *Fordiophyton*, and finally clade I (which includes *Bredia*, *Blastus*, and clade H, containing *Kerriothyrsus*, *Phyllagathis erecta*, and *Fordiophyton breviscapum*).

The species tree ST2 (Appendix S7) inferred from the 396‐locus data set receives less support (LPP < 0.9) for many nodes but is topologically similar to ST1, with some conflicting relationships. The first branching clade is also *Opisthocentra*, followed by *Boyania colombiana*, *Tryssophyton*, *Boyania kenwurdackii*, and *Phainantha*, then *Benna*. Within the afrotropical clade, relationships are mostly identical to ST1, except one species of *Gravesia* is placed elsewhere, nested within the Asian clade A. Within the primarily Asian clade, *Kendrickia* and *Sonerila* are successively sister to the rest, and clade A is recovered as sister to clade C with low support (LPP = 0.54). In contrast to ST1, clade D and *Styrophyton* are not resolved as monophyletic, with *Oxyspora* sp. ERR5084301 sequenced from PAFTOL failing to group with the other *Styrophyton* species. Additionally, some branches of ST2 are notably long, all of which are in the primarily Asian clade.

The species tree ST3 (Appendix S8), inferred using transcriptomic and genomic data, is topologically consistent with ST1.

### Gene tree conflicts

The QS analysis revealed abundant conflicts among gene trees, with branches having low local posterior probabilities, often with high discordance (Appendix S9). In the backbone, notable areas of low or counter‐support and high discordance include the Asian clade D (LPP = 0.79, QC/QD/QI = −0.014/0.78/0.2), and the clade comprising Asian clade E + *Phyllagathis rotundifolia + Oxyspora paniculata* + *Oxyspora* (Bornean) clade (LPP = 0.22, QC/QD/QI = 0.007/0.96/0.25). Within the identified major clades, many branches have high LPP but are weakly supported (QC < 0.2), particularly within the Asian clade.

## DISCUSSION

### An effective approach to integrating sequencing data from multiple sources

Efforts to integrate data from various sources, including target enrichment data, have primarily focused on recovering targeted loci from other data sets (Bossert et al., 2019; Wang et al., 2024). In this study, we compared using the Melastomataceae probe set alone for locus recovery with an approach that additionally used new reference sequences designed to capture all available data. The phylogenetic tree inferred from the Melastomataceae probe set loci was overall less well‐supported compared to the phylogenetic tree based on the data set acquired by using the new reference loci (Figure 5; Appendix S7). Several well‐established clades were not resolved as monophyletic in the former tree but were monophyletic in the latter.

Importantly, mapping data types onto the phylogeny showed that taxa sequenced with different strategies (target enrichment with Angiosperms353 probe set, Hyb‐Seq, DGS, RNA‐Seq, and annotated genomes) were interspersed across the tree rather than forming clades according to data type (Figure 5). This pattern supports the robustness of our integrative framework and suggests that heterogeneous genomic data can be reliably combined when appropriate reference construction and orthology inference are applied.

It has been suggested that increasing the number of gene trees (as has been done here) can improve the accuracy of species tree inference (Zhang et al., 2018), particularly under conditions of high incomplete lineage sorting (ILS) (Molloy and Warnow, 2017). It has also been mathematically demonstrated that the number of true gene trees required by ASTRAL to accurately reconstruct the species tree is proportional to the inverse of the shortest branch length (Shekhar et al., 2018). Given the high level of ILS observed in the Asian Sonerileae in the previous study (Zhou et al., 2022), it is likely that the phylogenetic informativeness of the Melastomataceae probe set is insufficient for certain parts of the tree. This limitation may be further exacerbated by the low on‐target rate of this probe set (Amarasinghe et al., 2021; Jantzen et al., 2022; Quakenbush et al., 2025), making the integrative approach employed here even more valuable.

The phylogenetic relationships within the Asian Sonerileae have long been unresolved, even when phylogenomic data were used (Baker et al., 2021; Maurin et al., 2021). In our study, most nodes of the Asian Sonerileae were resolved, consistent with the results of Zhou et al. (2022), in which 332 orthologs were obtained by sequencing a genome and several transcriptomes, followed by retrieving orthologs from genome resequencing data (DGS data) through read mapping. Our comparable results demonstrated that this more inclusive approach—which does not require genome sequencing and integrates diverse sequencing data sets, including target enrichment data—can produce accurate phylogenies while leveraging previously generated data rather than repeating the sequencing effort.

By including a large number of loci in the reference file, we increased the likelihood of maximizing the use of sequenced reads data. Nearly half of the Angiosperms353 loci (176 of 353) and more than half of the Melastomataceae probe set loci (221 out of 396) were found to match these orthologs (Appendix S2). The recovery was further improved by including the loci unique to the Melastomataceae probe set (i.e., those not overlapping with the new reference) in the phylogenetic analysis. For instance, *Gravesia setifera*, which was sequenced with the Angiosperms353 probe set, had not previously been grouped with other sampled *Gravesia* species (Baker et al., 2021; Maurin et al., 2021). In this study, represented by only two loci, *Gravesia* was placed within the primarily Asian clade in the phylogeny based on 396 loci; however, with 21 loci representing it, all sampled *Gravesia* species formed a monophyletic clade in the phylogeny using the new reference.

Notably, seven of 16 taxa sequenced using Angiosperms353 had more than 400 loci represented in the orthologs (Appendix S4), surpassing the number of targeted loci, suggesting that off‐target reads can be effectively utilized (Allio et al., 2020; Costa et al., 2021; Reichelt et al., 2021; Lagou et al., 2024). Additionally, we demonstrated that Hyb‐Seq and DGS can recover thousands of loci, with DGS performing slightly better (Hyb‐Seq: average 2774, median 3241; DGS: average 3738, median 3994). The sizes of the sequenced Melastomataceae genomes range from 246.4 to 299.8 Mb (Hao et al., 2022; Zhong et al., 2023; Huang et al., 2024), making it easy to achieve high coverage with genome sequencing, which may explain the effectiveness of Hyb‐Seq and DGS in this study. Since DGS does not require target enrichment, it also reduces the time and costs associated with designing and developing probe sets, extensive lab work, and chemicals. The method employed in this study offers a promising approach for integrating data generated from different sequencing strategies.

Despite its advantages, the integrative approach presented here also has several potential limitations. First, although this approach performed well for Melastomataceae, its applicability to lineages with much larger genomes remains to be tested. Secondly, integrating heterogeneous data sets inevitably results in uneven locus recovery and missing data across taxa, which may affect resolution or support at some nodes. Finally, the performance of the approach depends on the taxonomic coverage of the reference file, which requires access to transcriptomic/genomic resources. Although large‐scale genome sequencing projects such as 10KP (Cheng et al., 2018) and the Darwin Tree of Life (The Darwin Tree of Life Project Consortium, 2022) are rapidly increasing available resources, generating comprehensive coverage across many plant groups will take time.

### Phylogenetic relationships within Sonerileae

By integrating various types of sequencing data, we generated a well‐supported, near‐comprehensive, genus‐wide phylogeny for Sonerileae (Figure 5). This phylogeny is largely consistent with previous studies regarding the relationships among the African, Asian, and Neotropical clades (Baker et al., 2021; Zhou et al., 2022; Veranso‐Libalah et al., 2023) but provides greater resolution and improved sampling within those clades. The neotropical lineages and the African endemic *Benna* are successively sister to the rest of the tribe, which is further divided into a predominantly afrotropical clade and a primarily Asian clade.

The five sampled neotropical species from four genera appear as the first diverging lineages in the tribe. This pattern is similar to the results of Baker et al. (2021) and Veranso‐Libalah et al. (2023), although the exact branching order of these lineages differs. *Boyania* is recovered as nonmonophyletic, with *B. colombiana* sister to all Sonerileae except *Opisthocentra*, and *B. kenwurdacki* sister to *Phainantha laxiflora*. *Boyania* comprises three species and had already been shown to be nonmonophyletic based on Sanger data (Bacci et al., 2019). *Phainantha* has five recognized species, of which only two have been sequenced (one here), and they were resolved as sisters based on Sanger sequencing (Bacci et al., 2019; Wurdack and Michelangeli, 2019). *Boyania kenwurdackii* was represented by only 62 sequences and *P. laxiflora* by only 362. Incorporating more well‐sequenced neotropical taxa, including the unsampled monotypic genera *Neblinanthera* and *Tateanthus*, may help clarify the relationships of these early‐branching clades and assess whether gene flow contributed to the weak support.

The placement of the Sri Lankan endemic *Kendrickia* as sister to the afrotropical clade, first identified by Quakenbush et al. (2025), with *Kendrickia* represented by 255 loci, has been confirmed by our results with 3223 loci representing it. The internal relationships across different genera within the afrotropical clade are largely consistent with the Sanger‐sequence‐based phylogeny (Veranso‐Libalah et al., 2023), with the exceptions of the *Amphiblemma‐Cincinnobotrys‐Preussiella* clade and the placement of *Bourdaria*. In our phylogeny, *Amphiblemma* is sister to *Cincinnobotrys*, and *Bourdaria* is sister to the rest of the afrotropical clade, whereas in the Sanger phylogeny, *Amphiblemma* is sister to *Preussiella*, and *Gravesia*, instead of *Bourdaria*, is recovered as sister to the rest of the Afrotropical clade. Our results support the taxonomic treatments by Veranso‐Libalah et al. (2023), which separate *Mendelia* from *Amphiblemma* and *Bourdaria* from *Cincinnobotrys*.

Zhou et al. (2022) identified 34 lineages (potentially representing genera after taxonomic revision) and nine major clades within the remaining Asian Sonerileae. In this study, we sampled all of these lineages and also included new sequences for many fleshy‐fruited taxa. All 34 sampled lineages and the nine major clades were successfully recovered, corroborating the findings of Zhou et al. (2022). However, there are four discordant placements between our species tree (Figure 5) and the Astral coalescent tree from Zhou et al. (2022). These discrepancies involve the backbone of the Asian Sonerileae, the placement of *Oxyspora paniculata* and *Driessenia axantha*, and the placement and composition of clade F. In our phylogeny, clade C is the first branching clade of the Asian Sonerileae, followed by clade A and clade B, then the rest of the Asian Sonerileae. By contrast, Zhou et al. (2022) placed clade A as the first branching clade, followed by a clade comprising clade B and C, and then the rest of Asian Sonerileae. The very short branches along the backbone suggest a history of rapid radiation. Our results are consistent with the species tree based on transcriptomic and genomic data (Appendix S8). In our phylogeny, *Oxyspora paniculata* is sister to a clade comprising clade E and *Phyllagathis rotundifolia*, whereas Zhou et al. (2022) placed it as sister to clade F. The LPP support is weak, and the QS analysis shows counter‐support in both phylogenies (LPP = 0.83, QC/QD/QI = −0.016/0.77/0.26; LPP = 0.65, QC/QD/QI = −0.26/0.28/0.81). In our phylogeny, *Driessenia axantha* is nested within clade B, while in Zhou et al. (2022), it is placed as sister to clade B. In our analysis, the placement was well supported based on LPP but weakly supported based on the QC score (LPP = 1, QC/QD/QI = 0.015/0.95/0.25), whereas it was fully supported (LPP = 1, QC/QD/QI = 1/ − /1) in the phylogeny of Zhou et al. (2022). Furthermore, clade F of Zhou et al. (2022) comprises *Aschistanthera*, *Styrophyton*, and the *Oxyspora* Bornean clade. In contrast, our phylogeny does not place the Bornean *Oxyspora* clade within clade F; instead, it is recovered as sister to a clade comprising Asian clade E, *Phyllagathis rotundifolia*, and *Oxyspora paniculata*. This relationship, however, is weakly supported (LPP = 0.22; QC/QD/QI = 0.007/0.96/0.25).

The placements of the fleshy‐fruited taxa are consistent with those of Quakenbush et al. (2025). *Kendrickia* is sister to the afrotropical clade, and *Heteroblemma* and *Catanthera* form a clade that is sister to the *Phyllagathis* (thyrsoid) clade. The former genera *Pachycentria* and *Plethiandra* are nested within *Medinilla*. Within the highly diverse genus *Medinilla*, all 15 clades identified by Quakenbush et al. (2025) were recovered, with most internal relationships aligning closely. The exception is *Medinilla maidenii*, which in our phylogeny forms a clade with the *Medinilla* Western + Eastern Superclade with full support (LPP = 1), whereas Quakenbush et al. (2025) found it formed a clade with the *Medinilla* Western Superclade, albeit with low support (LPP = 0.65). Our QS results for the branch (QC/QD/QI = 0.00088/0.95/0.35) indicate rapid radiation, rather than gene flow. This case serves as an example of improving the accuracy of species tree inference by increasing the number of genes in the context of ILS.

## CONCLUSIONS

In this study, we demonstrated the potential of integrating various types of NGS data for phylogenetic analysis by constructing an inclusive reference from publicly available genomic and transcriptomic data. Applying this approach to Sonerileae resulted in a well‐represented and well‐supported phylogeny. For Sonerileae, this phylogenetic framework provides a basis for future systematic work, including improving sampling for unsampled and undersampled genera (e.g., the neotropical lineages), investigating the causes of gene tree conflicts, and integrating molecular and morphological evidence. Such efforts will be important for resolving the remaining taxonomic and phylogenetic uncertainties and for understanding trait evolution and biogeographic history of the tribe. This approach shows promise for unifying heterogeneous NGS data generated through different sequencing strategies and can be broadly applied to other groups facing similar challenges.

## AUTHOR CONTRIBUTIONS

L.C. conceptualized and designed the study, performed lab work and data analysis, and drafted the manuscript. M.C.V.L. conducted fieldwork, acquired funding, and provided supervision. J.P.Q. conducted fieldwork and lab work. Y.L. contributed key data. F.A.M. conducted fieldwork and provided supervision. D.F.M.B. assisted with data analysis. G.K. acquired funding and provided supervision. All authors contributed to revising the manuscript.

## Supporting information


**Appendix S1:** Detailed list of taxa sampled in this study, including voucher information for newly generated data and accession numbers for public data.

**Appendix S2:** Matching loci between the newly developed reference comprising 5626 loci and the Melastomataceae probe set containing 396 loci, and the Angiosperms353 probe set.

**Appendix S3:** Summary of sequence recovery across samples.

**Appendix S4:** Frequency of taxa in the final orthologs for the new reference data set. Taxa sequenced using the same strategy are additionally separately listed.

**Appendix S5:** Frequency of taxa in the final orthologs for the 396‐locus data set. Taxa sequenced using the same strategy are additionally separately listed.

**Appendix S6:** Distribution of orthologs per taxon by different sequencing methods in the final 225 orthologs from the 396‐locus data set.

**Appendix S7:** ASTRAL species tree ST2 inferred from 184 taxa and 225 orthologs from the 396‐locus data set, showing the relationships within Sonerileae. Local posterior probabilities (LPP) are shown above branches where LPP < 1.

**Appendix S8:** ASTRAL species tree ST3 inferred from 18 taxa and 5626 orthologs using only genomic and transcriptomic data, showing the relationships within Sonerileae. Local posterior probabilities (LPP) are shown above branches where LPP < 1.

**Appendix S9:** Gene tree conflicts measured with quartet sampling (QS) analysis. Each branch is annotated with three QS scores: quartet concordance (QC), quartet differential (QD), and quartet informativeness (QI).

## Data Availability

The newly developed reference, final sequence alignments, gene trees, and species trees generated in this study, as well as scripts used to run analyses are available at Zenodo (https://doi.org/10.5281/zenodo.19453003).
