# Supplementary material for: Integrative utilization of genomic resources for improved phylogenetic resolution in Sonerileae (Melastomataceae)
Source: Am J Bot. 2026 Jun 10;113(6):e70216. doi: 10.1002/ajb2.70216 (PMC13280967; doi:10.1002/ajb2.70216)

Appendix S6. Distribution of orthologs per taxon by different sequencing methods in the final 225 orthologs from the 396-locus data set. DGS, deep genome skimming.

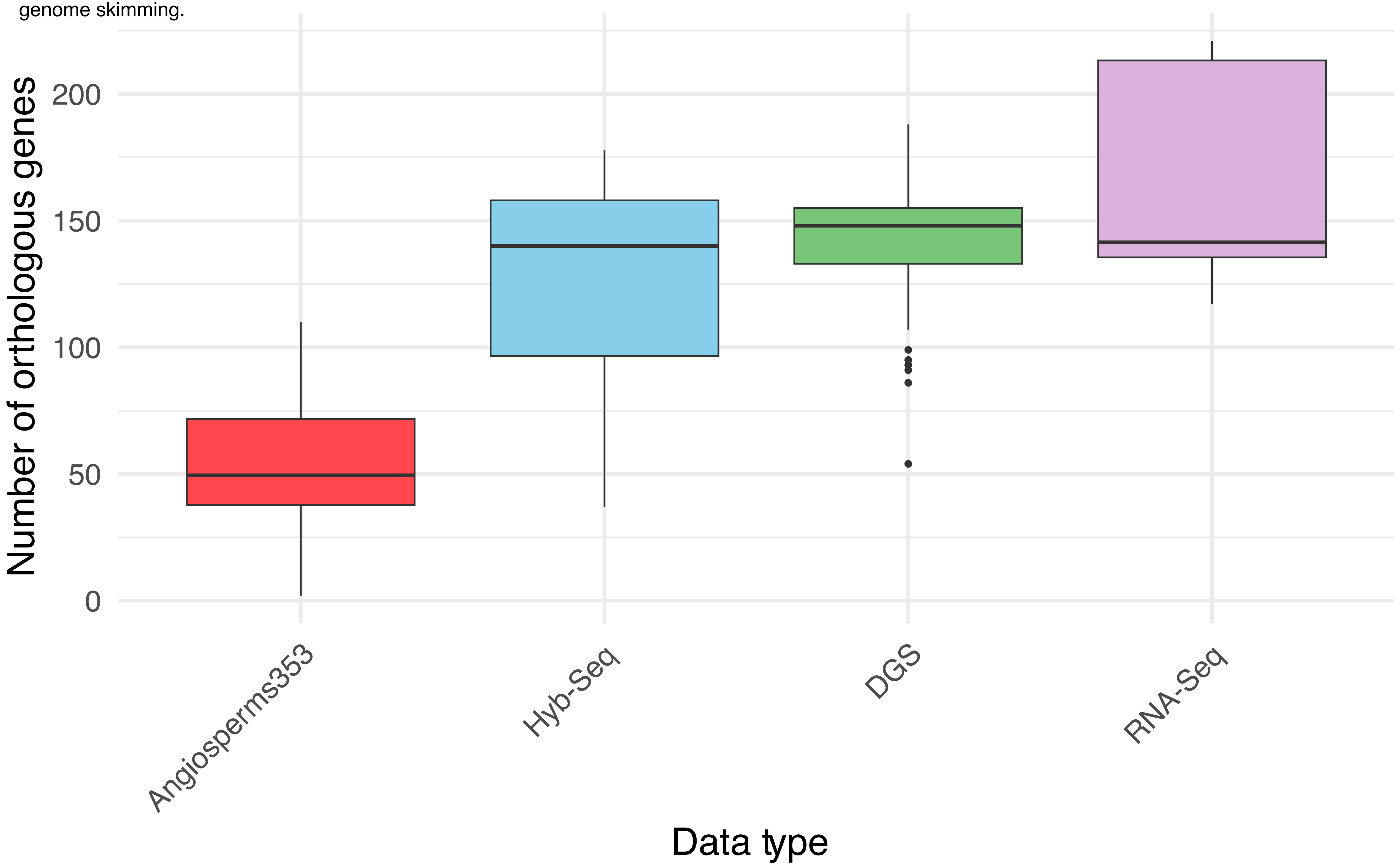

Supplement: Supplementary file 6 — Appendix S6: Distribution of orthologs per taxon by different sequencing methods in the final 225 orthologs from the 396‐locus data set. [file AJB2-113-e70216-s009.pdf]
