# Supplementary material for: Integrative utilization of genomic resources for improved phylogenetic resolution in Sonerileae (Melastomataceae)
Source: Am J Bot. 2026 Jun 10;113(6):e70216. doi: 10.1002/ajb2.70216 (PMC13280967; doi:10.1002/ajb2.70216)

Appendix S7. ASTRAL species tree ST2 inferred from 184 taxa and 225 orthologs from the 396-locus data set, showing the relationships within Sonerileae. Local posterior probabilities <1 are shown above branches. DGS, deep genome skimming.

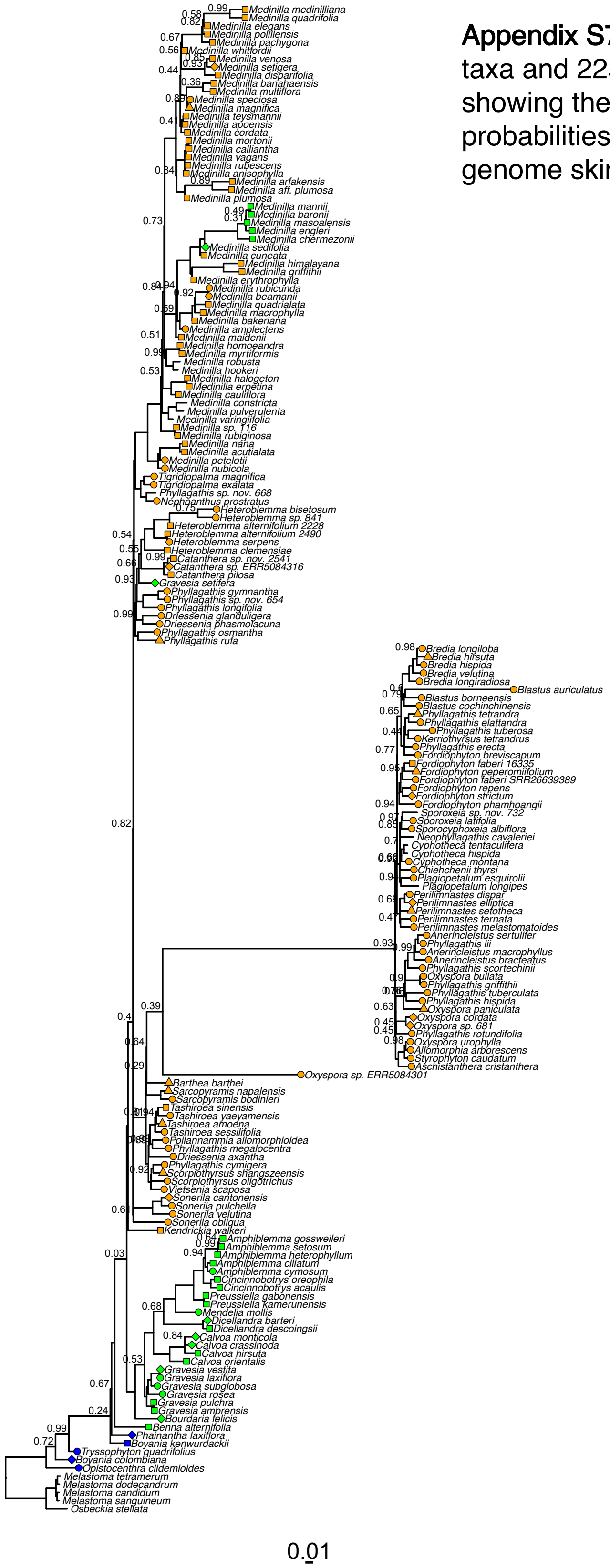

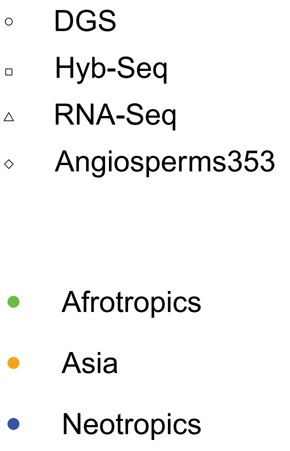

Supplement: Supplementary file 7 — Appendix S7: ASTRAL species tree ST2 inferred from 184 taxa and 225 orthologs from the 396‐locus data set, showing the relationships within Sonerileae. Local posterior probabilities (LPP) are shown above branches where LPP < 1. [file AJB2-113-e70216-s005.pdf]
