# Supplementary material for: Integrative utilization of genomic resources for improved phylogenetic resolution in Sonerileae (Melastomataceae)
Source: Am J Bot. 2026 Jun 10;113(6):e70216. doi: 10.1002/ajb2.70216 (PMC13280967; doi:10.1002/ajb2.70216)

Appendix S8. ASTRAL species tree ST3 inferred from 18 taxa and 5626 orthologs using only genomic and transcriptomic data, showing the relationships within Sonerileae. Local posterior probabilities <1 are shown above branches.

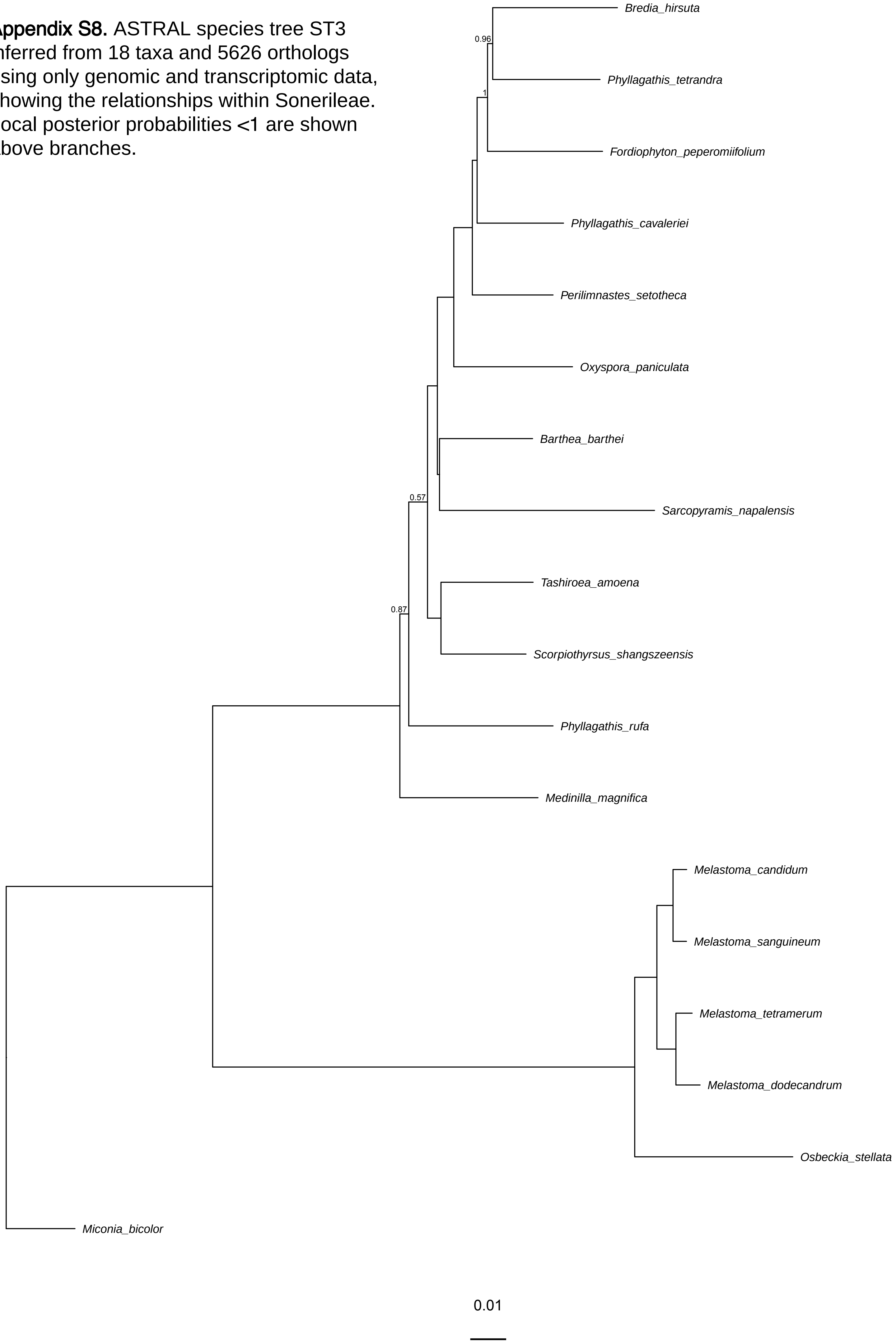

Supplement: Supplementary file 8 — Appendix S8: ASTRAL species tree ST3 inferred from 18 taxa and 5626 orthologs using only genomic and transcriptomic data, showing the relationships within Sonerileae. Local posterior probabilities (LPP) are shown above branches where LPP < 1. [file AJB2-113-e70216-s002.pdf]
