# Supplementary figures and images for: Integrative utilization of genomic resources for improved phylogenetic resolution in Sonerileae (Melastomataceae)
Source: Am J Bot. 2026 Jun 10;113(6):e70216. doi: 10.1002/ajb2.70216 (PMC13280967; doi:10.1002/ajb2.70216)

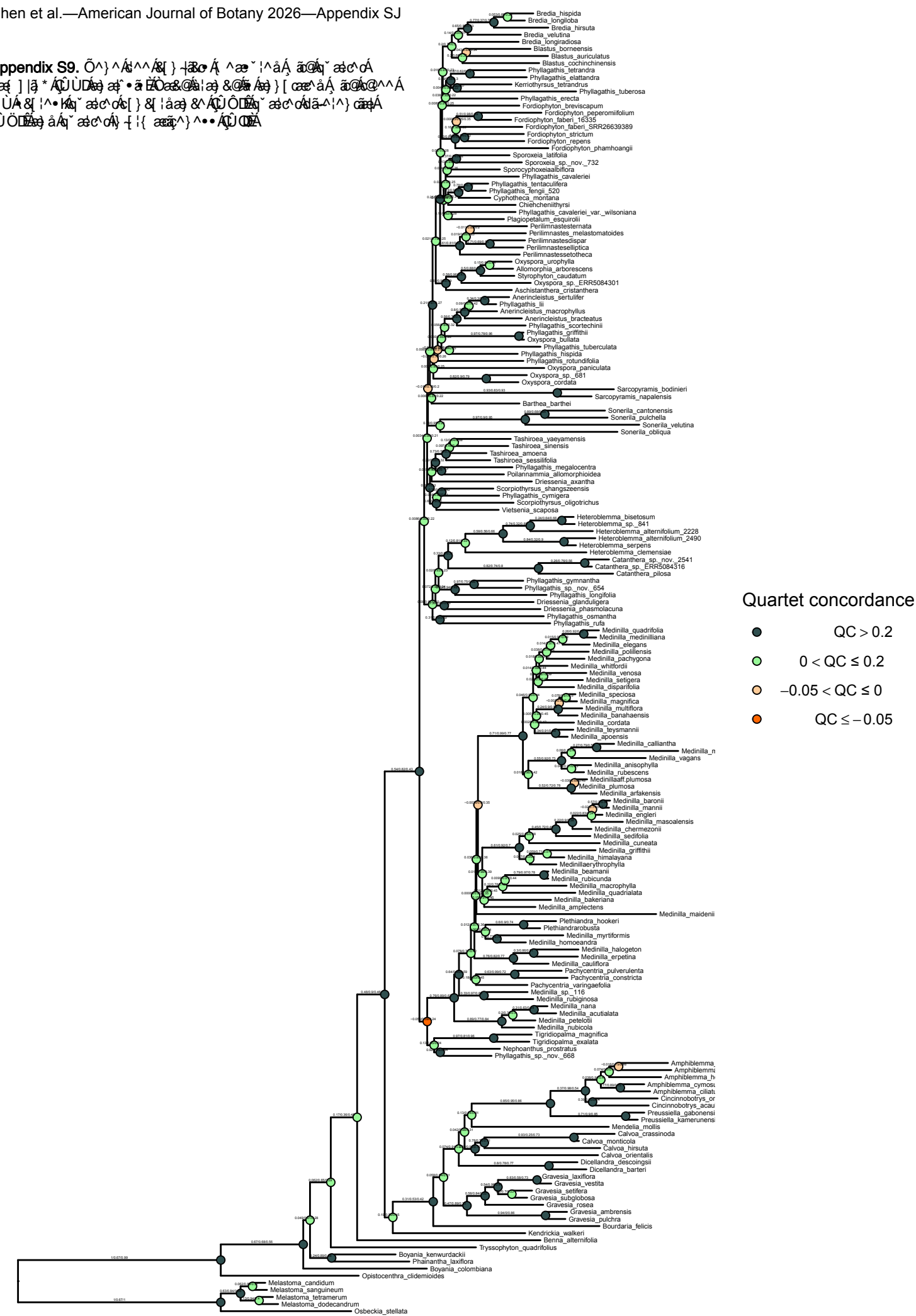

Supplement: Supplementary file 9 — Appendix S9: Gene tree conflicts measured with quartet sampling (QS) analysis. Each branch is annotated with three QS scores: quartet concordance (QC), quartet differential (QD), and quartet informativeness (QI). [file AJB2-113-e70216-s007.pdf]
